# Cloning, Functional Characterization and Nutritional Regulation of Δ6 Fatty Acyl Desaturase in the Herbivorous Euryhaline Teleost Scatophagus Argus

**DOI:** 10.1371/journal.pone.0090200

**Published:** 2014-03-03

**Authors:** Dizhi Xie, Fang Chen, Siyuan Lin, Shuqi Wang, Cuihong You, Óscar Monroig, Douglas R. Tocher, Yuanyou Li

**Affiliations:** 1 Marine Biology Institute of Shantou University & Guangdong Provincial Key Laboratory of Marine Biotechnology, Shantou, Guangdong, China; 2 Institute of Aquaculture, University of Stirling, Stirling, Scotland, United Kingdom; CNRS, France

## Abstract

Marine fish are generally unable or have low ability for the biosynthesis of long-chain polyunsaturated fatty acids (LC-PUFA) from C18 PUFA precursors, with some notable exceptions including the herbivorous marine teleost *Siganus canaliculatus* in which such a capability was recently demonstrated. To determine whether this is a unique feature of *S. canaliculatus* or whether it is common to the herbivorous marine teleosts, LC-PUFA biosynthetic pathways were investigated in the herbivorous euryhaline *Scatophagus argus*. A putative desaturase gene was cloned and functionally characterized, and tissue expression and nutritional regulation were investigated. The full-length cDNA was 1972 bp, containing a 1338 bp open-reading frame encoding a polypeptide of 445 amino acids, which possessed all the characteristic features of fatty acyl desaturase (Fad). Functional characterization by heterologous expression in yeast showed the protein product of the cDNA efficiently converted 18:3n-3 and 18:2n-6 to 18:4n-3 and 18:3n-6, respectively, indicating Δ6 desaturation activity. Quantitative real-time PCR showed that highest *Δ6 fad* mRNA expression was detected in liver followed by brain, with lower expression in other tissues including intestine, eye, muscle, adipose, heart kidney and gill, and lowest expression in stomach and spleen. The expression of *Δ6 fad* was significantly affected by dietary lipid and, especially, fatty acid composition, with highest expression of mRNA in liver of fish fed a diet with a ratio of 18:3n-3/18:2n-6 of 1.72:1. The results indicated that *S. argus* may have a different LC-PUFA biosynthetic system from *S. canaliculatus* despite possessing similar habitats and feeding habits suggesting that LC-PUFA biosynthesis may not be common to all marine herbivorous teleosts.

## Introduction

Long-chain polyunsaturated fatty acids (LC-PUFA), PUFA with chain lengths ≥C20 and ≥3 unsaturations, such as the arachidonic (ARA, 20:4n-6), eicosapentaenoic (EPA; 20:5n-3) and docosahexaenoic (DHA; 22:6n-3) acids, are essential components of biomembranes of all cells and tissues, and have crucial roles in growth, ontogenesis, reproduction, stress and immune responses as well as development of the nervous system [1], [2]. In addition, they also have key roles in the inflammatory response and consequently in several inflammatory and pathological conditions, including metabolic disorders, cardiovascular and neurological diseases [3]–[5]. As fish are the primary source in the human diet of the physiological-active n-3 LC-PUFA [6]–[8], the biosynthesis, metabolism and nutrition of LC-PUFA in fish have attracted increasing research interest.

The biosynthesis of LC-PUFA from α-linolenic (18:3n-3, LNA) and linoleic (18:2n-6, LA) acids requires a series of fatty acyl desaturase (Fads) and elongation of very long-chain fatty acids (Elovl) enzymes [9]. Fish species vary in their capacity to biosynthesize LC-PUFA depending on their complement of these key enzymes [1], [10]. Freshwater species, such as carp, tilapia, and trout, possess the capacity to synthesize LC-PUFA from precursor C18 PUFA [1], [11]. In contrast, marine fish have generally been regarded as species with limited capability for endogenous synthesis of LC-PUFA from their C18 precursors, and hence have a strict dietary requirement for LC-PUFA [1], [12]. The high availability of C20–22 LC-PUFA in marine food webs and the resultant low selection pressure was speculated to have resulted in the loss of specific enzymatic activities of the LC-PUFA biosynthetic pathway in marine fish species [13]. Recently, however, we reported that the herbivorous marine fish *Siganus canaliculatus*, or rabbitfish, could grow well without dietary LC-PUFA [14], [15]. In addition, we recently isolated two desaturases with Δ4 and Δ6/Δ5 specificities, and two elongases (Elovl4 and Elovl5) from *S. canaliculatus* [16], [17], which suggested that rabbitfish would have the ability to endogenously synthesize LC-PUFA. Thus, rabbitfish became the first marine teleost in which all the enzymatic activities required for the production of LC-PUFA from C18 PUFA were demonstrated. The effect of ‘trophic level’, the position of an organism in the food web, was thus hypothesized as a potential further factor influencing the LC-PUFA biosynthetic capability of teleost fish in addition to the above mentioned environmental factors.

*Scatophagus argus* has similar habitat and feeding habits to rabbitfish, and is distributed widely in freshwater, brackish and marine habitats of the Indo-Pacific, South and South East Asia, and is also a herbivore that feeds on largely plant material [18]–[20]. In order to investigate whether *S. argus* has an enzymatic complement for LC-PUFA biosynthesis similar to that of rabbitfish, we aimed to isolate and characterize genes encoding Fads and Elovl enzymes with putative roles in the pathways. In the present study, we report the cloning, functional characterization, tissue expression and nutritional regulation of a Fads2-like desaturase from *S. argus*. The results have increased our knowledge of the relationships between LC-PUFA biosynthesis pathways and the habitat and feeding habit of fish species, and provide the basis for studying the regulatory mechanisms controlling LC-PUFA biosynthesis pathways in *S. argus*.

## Materials and Methods

### Diets, fish, feeding trial and sampling

With casein as protein source, and fish oil (rich in LC-PUFA) or soy oil and perilla oil (both LC-PUFA-free) as lipid sources, six iso-proteic and iso-lipidic experimental diets (D1-D6) were formulated with 32% crude protein and 8% crude lipid. Diet D2 contained fish oil (FO) as control, and diets D1, D3-D6 contained different proportions of soybean oil and perilla oil, which resulted in ratios of LNA: LA of 0.14, 0.57, 0.84, 1.72 and 2.85, respectively. The dietary formulations, proximate and fatty acid compositions are shown in Table 1.

**Table 1:** Formulation and composition of the experimental diets.

		D1	D2	D3	D4	D5	D6
Dietary prescription(g/100 g diet)							
Casein	40.00		40.00	40.00	40.00	40.00	40.00
α- starch	5.00		5.00	5.00	5.00	5.00	5.00
Starch	34.40		34.40	34.40	34.40	34.40	34.40
Cellulose	9.00		9.00	9.00	9.00	9.00	9.00
Mineral premix^1^	1.00		1.00	1.00	1.00	1.00	1.00
Vitamin premix^2^	1.00		1.00	1.00	1.00	1.00	1.00
Others^3^	1.60		1.60	1.60	1.60	1.60	1.60
Fish oil	0.00		8.00	0.00	0.00	0.00	0.00
Perilla oil	0.00		0.00	5.68	3.84	5.84	8.00
Soybean oil	8.00		0.00	2.32	4.16	2.16	0.00
Proximate composition (%, dry matter basis)							
Moisture		11.49	12.39	11.38	9.89	10.29	12.07
Protein		33.21	32.59	31.92	32.83	32.74	32.63
Lipid		8.46	8.61	7.94	8.20	8.75	8.32
Ash		5.75	5.36	4.97	5.29	5.18	5.42
Main fatty acids (% area)							
18:2n6		43.09	2.87	37.47	25.24	22.39	16.53
18:3n6		0.79	0.26	0.73	0.94	0.88	0.90
18:3n3		7.40	1.41	21.67	21.92	39.72	59.75
20:4n6		/4	0.30	/	/	/	/
20:4n3		/	0.10	/	/	/	/
20:5n3		/	2.97	/	/	/	/
22:5n3		/	0.14	/	/	/	/
22:6n3		/	3.56	/	/	/	/
∑Saturated		24.13	55.93	19.02	28.07	21.90	23.28
∑Monoenes		24.32	27.96	20.04	22.61	22.04	19.54
∑n-6 PUFA		43.88	4.25	38.2	26.18	23.27	17.43
∑n-3 PUFA		7.40	8.19	21.67	21.92	39.72	49.75
LNA/LA		0.14	0.49	0.57	0.84	1.72	2.85

1 The amounts of following ingredients in per kg of premix are: iron, 8 g; molybdenum, 1 g; zinc, 30 g; manganese, 2 g; cobalt, 1 g; iodine, 500 mg; selenium, 40 mg.

2 The amounts of following vitamins in per kg of premix are: A, 4×106 IU; D3, 2×106 IU; E, 60 g; K3, 6 g; B1, 7.5 g; B2, 16 g; B6, 12 g; B12, 100 mg; nicotinic acid, 88 g; pantothenic acid, 36 g; folic acid, 2 g; biotin, 100 mg; inositol, 100 g; C-monophopholipid, 200 g.

3 The amounts of following ingredients in per 100 g of diet are: CaHPO4, 0.05 g; Methionine, 0.05 g; Lycine, 0.05 g; Choline chloride, 0.08 g; Vitamin C, 0.02 g.

4 Undetectable.

*S. argus* juveniles (body mass around 4.3 g) were purchased from a local hatchery, and reared in floating cages (0.6×0.6×3.0 m) located on the coast near Nan Ao Marine Biology Station (NAMBS), Shantou University and fed an equal mix of the six experimental diets for two weeks before the start of feeding trial. The feeding trial was conducted in 18 floating cages (0.6×0.6×3.0 m) at ambient temperature, salinity and photoperiod, with each cage containing 25 fish that were allocated randomly. Fish in triplicate cages were fed one of the experimental diets twice a day (at 9:00 and 16:00 h) at 1–2% of body weight for 8 weeks. At the end of the feeding trial, fish were anaesthetized with 0.01% 2-phenoxyethanol (Sigma-Aldrich Inc., USA), and livers were collected from six fish per dietary treatment (2 fish per replicate cage) to investigate the effects of dietary fatty acid composition on gene expression. Livers were immediately frozen in liquid nitrogen and stored at −80°C prior to the analysis of desaturase mRNA expression by quantitative PCR (qPCR). In order to determine the tissue distribution of *S. argus* putative desaturase, eye, brain, liver, muscle, heart, gills, spleen, kidney and intestine were collected from six *S. argus* individuals (80–90 g) captured from the coast near NAMBS, after the fish were anaesthetized with 0.01% 2-phenoxyethanol. Tissue samples were frozen in liquid nitrogen immediately after collection and stored at −80°C until RNA extraction. All experiments and procedures were carried out according to the “Regulations for the Administration of Affairs Concerning Experimental Animals” established by the Guangdong Provincial Department of Science and Technology on the use and care of experimental animals. The study was reviewed and approved by the Ethics Committee of Animal Experiments of Shantou University.

### Molecular cloning of *Scatophagus argus* desaturase cDNA

Total RNA was extracted from *S. argus* liver using Trizol reagent (Invitrogen, USA) and reverse transcribed into cDNA using random primers and an appropriate RT-PCR kit (Invitrogen, USA). Degenerate primers (SaF1 and SaR1) were designed on the basis of highly conserved regions of desaturase genes of other fish species available in the GenBank database including rabbitfish (ABR12315), zebrafish (*Danio rerio*) (AAG25710) and gilthead seabream (*Sparus aurata*) (ADD50000), and used for amplifying partial fragments of *S. argus* putative desaturase cDNA (Table 2). PCR consisted of an initial denaturation at 94°C for 3 min, 35 cycles of denaturation at 94°C for 30 s, annealing at 57°C for 30 s and extension at 72°C for 1 min, followed by a final extension at 72°C for 10 min. The gel purified PCR products were cloned into the pMD18-T vector (Takara) for further sequencing (Sangon Biotechnology Company, Shanghai, China). Gene-specific primers (SaF2 and SaF3, SaR2 and SaR3) were then designed to produce the full-length desaturase cDNA by 5′ and 3′ rapid amplification of cDNA ends (RACE) PCR (GeneRacer kit, Invitrogen, USA) (Table 2).

**Table 2:** Sequences of primers used for cDNA cloning or determining mRNA content of *S. argus fads2*.

Primers for partial cDNA cloning	
SaF1	5′-ACCTGGGCCACATCCTGCT-3′
SaR1	5′-CCAGGCCAGATCCACCCAGT-3′
Primers for 5′RACE	
SaR2	5′-TGCTTGCGTAAAGTGCGGAAATCCTGTA-3′
SaR3	5′- AGCAAGATCGCACACAGAAGCGTCAG-3′
Primers for 3′RACE	
SaF2	5′-CGCTTCTGTGTGCGATCTTGCTGAC-3′
SaF3	5′-TTCCAGCATCACGCTAAACCCAACATCT-3′
Primers for ORF cloning	
SaF4	5′-CCCGGATCCAGGATGGGAGGTGGAGGCCAC-3′
SaR4	5′-CCGTCTAGATCATTTATGGAGATATGC-3′
Primers for real-time quantitative PCR	
fads2	
fadF	5′-GCTTCTGTGTGCGATCTTGC-3′
fadR	5′-GATGTTGGGTTTAGCGTGATGC-3′
18S rRNA	
18SF	5′-CGCCGAGAAGACGATCAAAC-3′
18SR	5′-TGATCCTTCCGCAGGTTCAC-3′

### Sequence and phylogenetic analysis of *S. argus* desaturase

The deduced amino acid (aa) sequence of the newly cloned putative desaturase gene from *S. argus* was aligned with other orthologues from human (*Homo sapiens*) (AAD20018), mouse (*Mus musculus*) (AAD20017), zebrafish (AAG25710), and rabbitfish (ABR12315) using Clustal W (www. ebi.ac.uk/tools/msa/clustalW2) [21]. The aa sequence identities of *S. argus* putative desaturase was compared to those of orthologues from other fish and mammals using the EMBOSS Needle Pairwise Sequence Alignment tool (http://www.ebi.ac.uk/Tools/psa/emboss_needle/). Phylogenetic analysis of desaturase polypeptides was performed by constructing a tree using the neighbor-joining method [22]. Confidence in the resulting phylogenetic tree branch topology was measured through bootstrapping through 10000 iterations.

### Functional characterization of putative desaturase by heterologous expression in yeast

Functional characterization of *S. argus* putative desaturase gene was conducted by expressing the PCR fragment corresponding to the open reading frame (ORF) in the yeast *Saccharomyces cerevisiae*. Primers SaF4 and SaR4 containing restriction sites for *Bam*HI and *Xba*I, respectively (underlined in Table 2), were designed for amplification of ORF from liver cDNA using the high fidelity *Pfu* DNA polymerase (TianGen, Beijing, China) under the following conditions: initial denaturation at 95°C for 3 min, followed by 35 cycles of denaturation at 95°C for 30 s, annealing at 68°C for 30 s and extension at 72°C for 90 s, with a final extension at 72°C for 10 min. The DNA fragment was purified and digested with the corresponding restriction endonucleases (New England Biolabs, UK) and ligated into the yeast episomal plasmid pYES2 (Invitrogen, USA). The plasmid construct pYES2-fads was used to transform *S. cerevisiae* (strain INVSc1, Invitrogen) using the S.C. Easy Comp Transformation kit (Invitrogen, USA). A single colony containing the desaturase construct was grown on *S. cerevisiae* minimal medium minus uracil (SCMM^-uracil^). Each culture was supplemented with one of the following fatty acid substrates: LNA (18:3n-3), LA (18:2n-6), eicosatetraenoic acid (20:4n-3), dihomo-γ-linolenic acid (20:3n-6), docosapentaenoic acid (22:5n-3), or docosatetraenoic acid (22:4n-6) obtained from Cayman Chemicals Co (Ann Arbor, MI, USA). The PUFA substrates were added at final concentrations of 0.5 (C18), 0.75 (C20) and 1.0 (C22) mM as uptake efficiency decreases with increasing chain length [16]. After culture for two days, yeast cells were harvested and washed as described previously for lipid extraction [16].

### Lipid extraction and fatty acid analysis

Yeast samples were homogenized in chloroform/methanol (2∶1, v/v) containing 0.01% 2,6-butylated hydroxytoluene (BHT) as antioxidant, and total lipid was extracted according to Folch et al. [23]. Fatty acid methyl esters (FAME) were prepared by transesterification with boron trifluoride etherate (ca. 48%, Acros Organics, NJ, USA) as described previously [14]. FAME were purified by TLC, resuspended in hexane [24], and separated using a gas chromatograph (GC2010-plus, Shimadzu, Japan) as described in detail previously [16]. Desaturase activity was calculated as the proportion of substrate fatty acid converted to desaturated fatty acid product as follows: 100× [product area/(product area + substrate area)].

### Analysis of desaturase expression by quantitative PCR

The mRNA levels of cloned putative desaturase gene in different tissues of *S. argus* or in livers of fish fed with diets D1–D6 for eight weeks were measured by quantitative real-time PCR (qPCR). Total RNA was extracted from tissues using Trizol reagent (Invitrogen, USA), and 1 µg total RNA was reverse transcribed into cDNA using AMV First-Strand cDNA Synthesis Kit (Invitrogen, USA). The mRNA levels of *S. argus* putative desaturase and the reference gene 18S rRNA in tissues were then quantified by qPCR (SYBR Green II) with primer-pairs fadF-fadR and 18SF-18SR, respectively (Table 2). Assays were run on an ABI Prism 7300 sequence detection system (PE Applied Biosystems, Foster City, CA) under the following conditions: samples were heated for 10 s at 95°C, and amplified for 40 cycles at 90°C for 5 s, and with a final step at 60°C for 31 s. The mRNA levels of *S. argus* putative desaturase in each sample were normalized relative to the expression of 18S rRNA calculated by the comparative threshold cycle (Ct) method [25].

### Statistics

Comparisons amongst treatments were analyzed by one-way analysis of variance (ANOVA) at a significance level of 0.05 following confirmation of normality and homogeneity of variance tests. The statistical analyses were computed using SPSS v17.0 (SPSS Inc., Chicago, IL, USA).

## Results

### Sequence and phylogenetic analyses of *S. argus* putative desaturase

**Figure 1:**
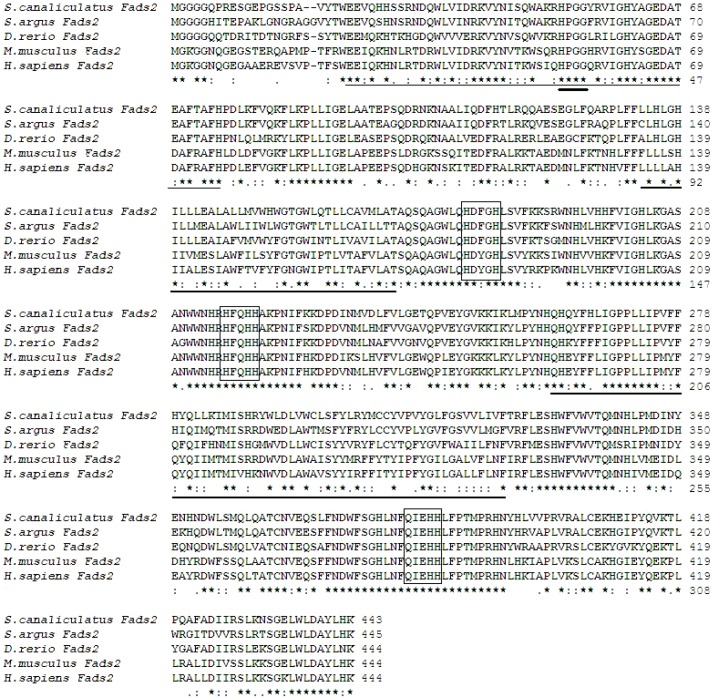
Alignment of *Scatophagus argus* Δ6 desaturase peptide sequence with those of *Siganus canaliculatus* (Fads2) (ABR12315), *Danio rerio* (Fads2) (AAG25710), *Mus musculus* (Fads2) (AAD20017) and *Homo sapiens* (Fads2) (AAD20018) using ClustalW. Identical and similar residues are marked with ‘*’ and ‘:’, respectively. The cytochrome-b5 like domain is underlined with a fine line and the heme-binding motifs with a short bold lines. The long bold underlines denote the trans-membrane regions, and the three histidine boxes are highlighted with frames.

**Figure 2:**
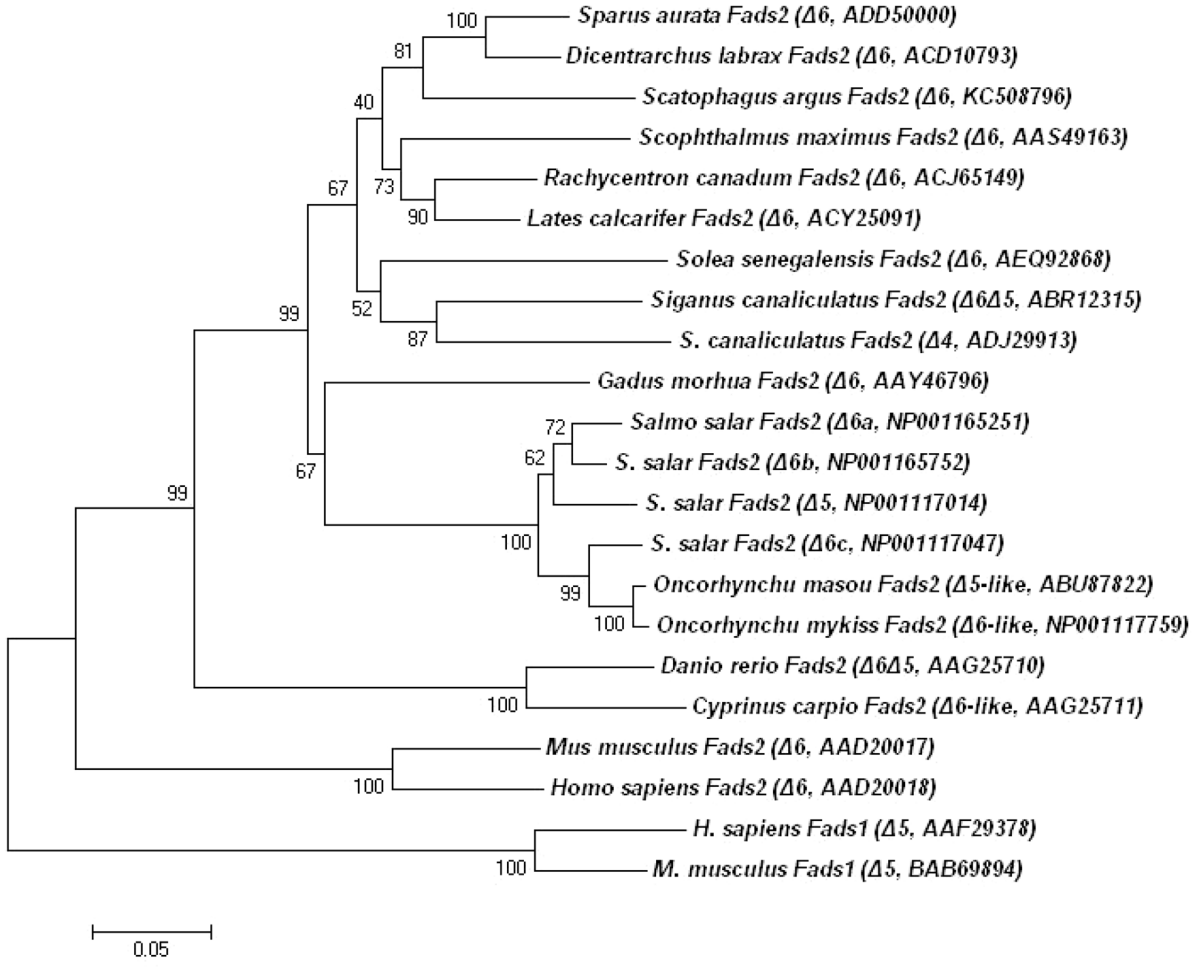
Phylogenetic tree comparing the deduced amino acids of *Scatophagus argus* Δ6 desaturase with mammals and other teleost homologs. The tree was constructed using the neighbor-joining method [55] with MEGA4. The horizontal branch length is proportional to the substitution rate per site. Numbers represent the frequencies with which the tree topology presented was replicated after 10000 bootstrap iterations.

The cloned *S. argus* putative desaturase cDNA was 1972 bp in full-length, which contained a 1338 bp ORF specifying a peptide of 445 aa. The sequence was deposited in GenBank database under the accession number KC508796. The deduced polypeptide sequence contained all the characteristics of microsomal fatty acyl front-end desaturases including two trans-membrane regions, three histidine-rich boxes, and an N-terminal cytochrome b5 domain containing the heme-binding motif HPGG (Figure 1). When compared to other vertebrate desaturase sequences, the *S. argus* putative desaturase showed 65% and 90–95% identity to those of mammals, and zebrafish and rabbitfish, respectively. Phylogenetic analysis comparing a variety of desaturases from fish, mammals, fungus and nematode, showed that all fish desaturase genes, including that for *S. argus*, clustered together and closer to mammalian Δ6 desaturases (Fads2) rather than to Δ5 desaturases (Fads1). In addition, the *S. argus* desaturase clustered most closely to gilthead sea bream and European sea bass (*Dicentrarchus labrax*) desaturases with Δ6 desaturation activity (Figure 2).

### Fatty acyl substrate specificity of the *S. argus* putative desaturase

**Figure 3:**
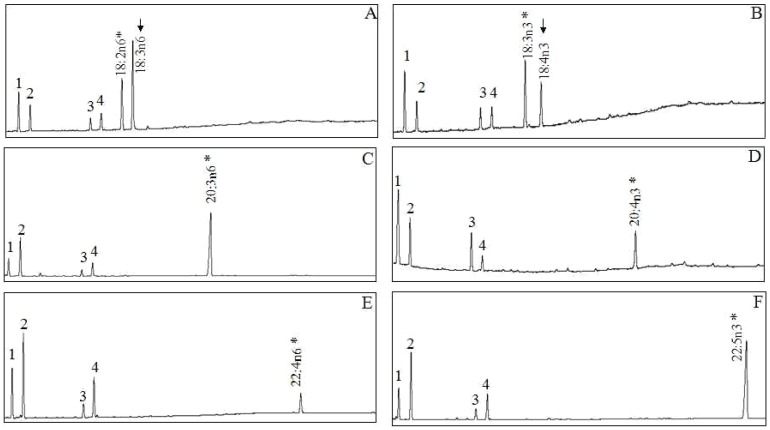
Functional characterization of *Scatophagus argus* putative desaturase in yeast *Saccharomyces cerevisiae*. FAME were extracted from yeast transformed with the pYES2-fads, and grown in the presence of FA substrates (*) 18:2n-6 (A), 18:3n-3 (B), 20:3n-6(C), 20:4n-3 (D), 22:4n-6 (E) and 22:5n-3 (F). Peaks 1–4 represent the main endogenous FAs of *S. cerevisiae*, namely 16:0, 16:1 isomers, 18:0 and 18:1n-9, respectively. Based on retention times, additional peaks (arrowed) were identified as 18:3n-6 (A) and 18:4n-3 (B). Vertical axis, FID response; horizontal axis, retention time.

The fatty acid specificity of the newly cloned *S. argus* putative desaturase was identified by heterologous expression in yeast *S. cerevisiae*. Fatty acid profiles of control yeast transformed with pYES2 vector alone were characterized by having only the four main endogenous fatty acids, namely 16:0, 16:1n-7, 18:0 and 18:1n-9 (peaks 1-4 in Figure 3), together with any exogenously added substrate fatty acid (data not shown). This is in agreement with *S. cerevisiae* having limited PUFA metabolism, including completely lacking endogenous enzymatic capabilities for PUFA substrates [16]. In the yeast transformed with pYES2-fads, however, additional peaks in yeast grown in the presence of LA (18:2n-6) and LNA (18:3n-3) were identified as 18:3n-6 (Figure 3A) and 18:4n-3 (Figure 3B), respectively, on the basis of GC retention time. These results indicated that the *S. argus* putative desaturase was a Fads2 with Δ6 desaturation activity. The conversion rates of 18:2n-6 to 18:3n-6 and 18:3n-3 to 18:4n-3 were approximately of 61% and 82%, respectively (Table 3). Transgenic yeast expressing the *S. argus* putative desaturase were unable to desaturate any of the other fatty acid substrates assayed (namely, 20:3n-6, 20:4n-3, 22:4n-6 and 22:5n-3), indicating it was unable to perform desaturations at the Δ5 and Δ4 positions (Figure 3, panels C–F).

**Table 3:** Substrate conversions of pYES-fads transformed yeast grown in presence of Δ6 (18:3n-3 and 18:2n-6), Δ5 (20:4n-3 and 20:3n-6) and Δ4 (22:5n-3 and 22:4n6) fatty acid (FA) substrates.

FA substrate	Product	Conversion (%)	Activity
18:3n-3	18:4n-3	82.25	Δ6
18:2n-6	18:3n-6	61.18	Δ6
20:4n3	20:5n-3	0.00	Δ5
20:3n6	20:4n-6	0.00	Δ5
22:5n3	22:6n-3	0.00	Δ4
22:4n6	22:5n-6	0.00	Δ4

Results are expressed as a percentage of total FA substrate converted to desaturated products.

### Tissue distribution of the *S. argus fads2*

**Figure 4:**
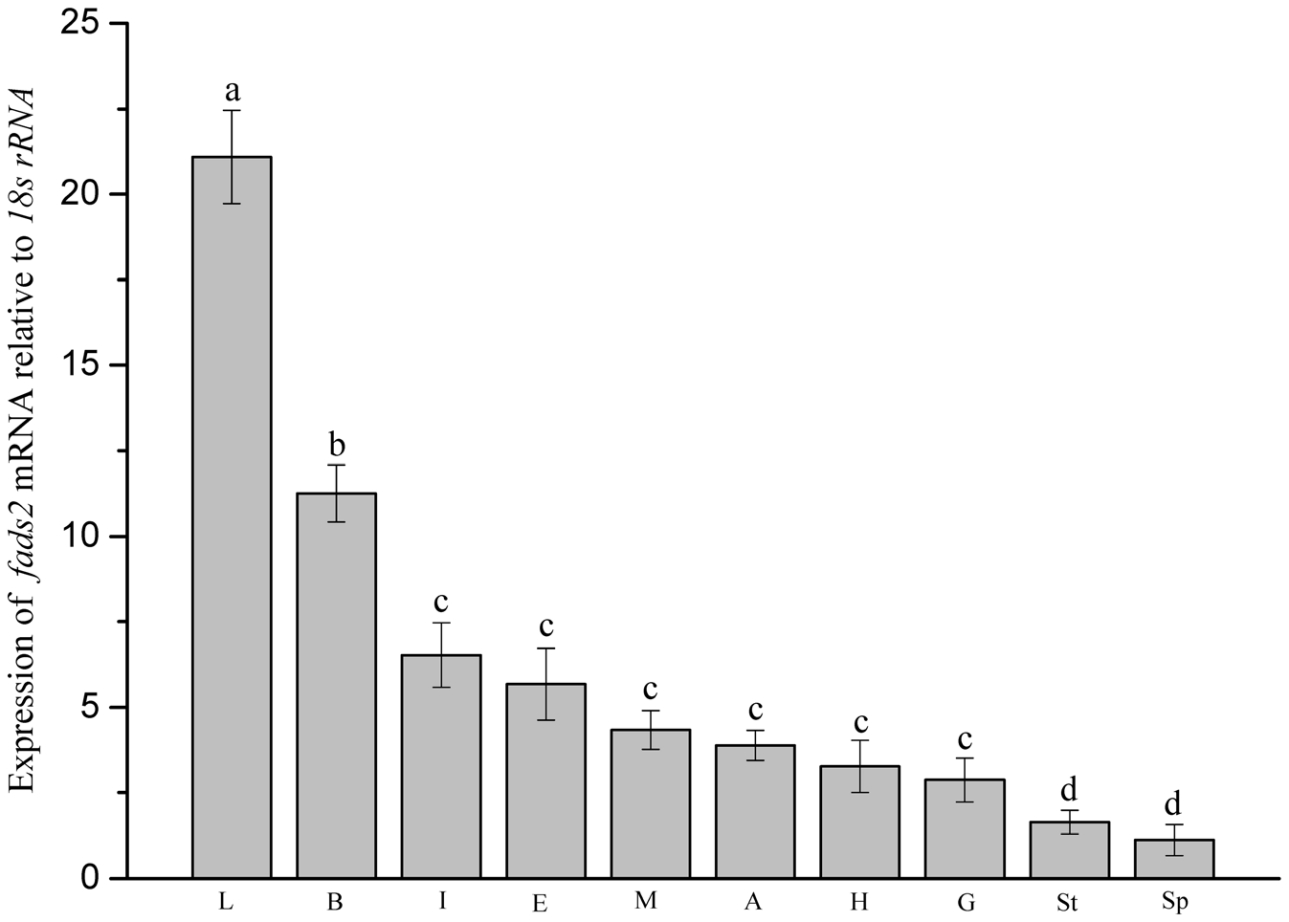
Relative expression levels of *fads2* mRNA in different tissues of *Scatophagus argus*. Expression values were normalized to those of *18S rRNA*. Data are means ± SEM (n = 6). Bars with different superscripts are significantly different (*P*<0.05, one-way ANOVA test). L, liver; B, brain; I, intestine; E, eye; M, muscle; A, adipose; H, heart; G, gill; St, stomach; Sp, spleen.

The expression of *S. argus* desaturase (*fads2*) was detected in all tissues examined with significantly highest expression in liver (*P*<0.05), followed by brain. Other tissues including intestine, eye, muscle, adipose, heart and gill showed relatively low expression levels, with stomach and spleen having the lowest expression signals (Figure 4).

### Effects of dietary fatty acid composition on expression of *fads2* mRNA in liver of *S. argus*

**Figure 5:**
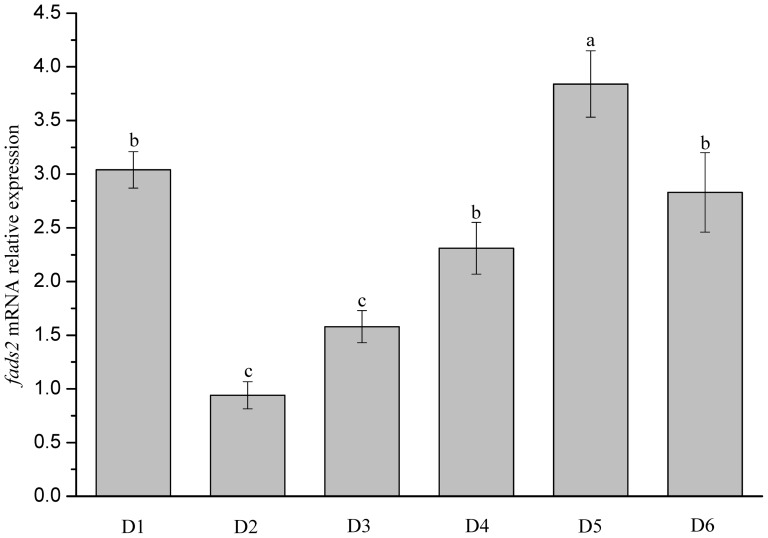
Relative expression levels of *fads2* mRNA in livers of *Scatophagus argus* fed six experimental diets. Expression values were normalized to those of *18S rRNA*. Data are means ± SEM (n = 6). Bars with different superscripts are significantly different (*P*<0.05, one-way ANOVA test). D2: control diet with fish oil as lipid source; D1, D3–D6: diets with blended vegetable oils as lipid source with dietary LNA/LA ratios of 0.14, 0.57, 0.84, 1.72, and 2.85, respectively.

In comparison with the expression level in liver of fish fed the control diet D2 with fish oil (FO), the mRNA levels of *fads2* were significantly higher in livers of fish fed diets D1, D3–D6 formulated with vegetable oil (VO), which suggested that the expression of *fads2* was up-regulated in fish fed diets containing reduced levels of LC-PUFA (Figure 5). More specifically, the highest expression level was detected in liver of fish fed diet D5, where the LNA/LA ratio was 1.72. Those in livers of fish fed diets D1 (LNA/LA = 0.14), D6 (LNA/LA = 2.85) and D4 (LNA/LA = 0.84) were all significantly higher compared to fish fed diet D2. In contrast, no significant difference was found between D3 (LNA/LA = 0.57) and D2 dietary group.

## Discussion

In the present study, the cDNA for a *fads2* gene was cloned from *S. argus*. In mammals, Fads2 catalyzes the first (Δ6) desaturation step in the LC-PUFA biosynthesis pathway from the C18 PUFA, LNA and LA, and it has been regarded as the rate-limiting enzyme in the pathway to EPA and ARA, respectively [26]. Mammalian Fads2 is entirely associated with Δ6 specificity, however, Fads2 in teleost species display considerable functional diversification having evolved to have a wide range of substrate specificities [27]. To date, Fads2 enzymes with Δ6 desaturase activities have been identified from freshwater fish including common carp (*Cyprinus carpio* var. Jian) [11] and Nile tilapia (*Oreochromis niloticus*) [28], diadromous species such as Atlantic salmon (*Salmo salar*) [29], masu salmon (*Oncorhynchus masou*) [30] and meagre (*Argyrosomus regius*) [31], and marine fish including gilthead seabream [32], Atlantic cod (*Gadus morhua*) [33], cobia (*Rachycentron canadum*) [34], European sea bass [35], Asian sea bass (*Lates calcarifer*) [36], nibe croaker (*Nibea mitsukurii*) [37], Atlantic bluefin tuna (*Thunnus thynnus*) [38], striped snakehead fish (*Channa striata*) [39] and black seabream (*Acanthopagruss chlegeli*) [40]. In addition, a further Fads2 from Atlantic salmon was shown to be a monofunctional Δ5 desaturase [41], and bifunctional Δ6/Δ5 Fads2 enzymes were reported in the freshwater species zebrafish (*Danio rerio*) [42] and marine species rabbitfish [16]. Thus, in addition to expressing a Δ4-desaturase, rabbitfish was the first marine fish in which a capability for Δ5-desaturation was demonstrated, as this enzymatic activity was previously believed to be generally absent in marine teleosts [13]. With respect to the noteworthy differences in desaturation activities among teleost Fads2, Castro et al [27] hypothesized that the losses and diversifications of crucially important genes in fatty acid metabolism during fish evolution might be linked to habitat-specific food web characteristics, such as LC-PUFA availability, in different environments.

The rabbitfish *S. canaliculatus* was the first marine teleost species reported to have the capability of LC-PUFA biosynthesis from C18 PUFA precursors and all the genes encoding key enzymes for LC-PUFA biosynthesis have been characterized including Fads2 desaturases with both Δ4 and Δ6/Δ5 activities, and Elovl5 and Elovl4 elongases capable of elongating C18, C20 and C22 PUFA [14], [16], [17]. To a large extent *S. argus* shares similar feeding habits (naturally feeding on algae and other plant material) and habitats (marine and brackish waters) to those of rabbitfish. However, as it has a monofunctional Δ6 desaturase, it does not appear to possess a similar LC-PUFA biosynthesis system with rabbitfish, which has a bifunctional Δ6/Δ5 desaturase and a monofunctional Δ4 desaturase [16]. Attempts have been made to clone further desaturases from *S. argus* and, although these have so far been unsuccessful, it is not possible to unequivocally discount the possibility that other fatty acyl desaturases, with activities other than Δ6, exist in the *S. argus* genome.

The *S. argus* Fads2, like the majority of Fads2 desaturases isolated from marine fish [32]–[40], has been characterized as displaying monofunctional Δ6 desaturation activity. Although phylogenetic analysis showed that the *S. argus* desaturase clustered closely with desaturases from carnivorous *S. aurata* and *D. labrax*, which was consistent with their measured activities, phylogeny and functionality do not necessarily correlate, at least when comparisons are made within teleosts. Atlantic salmon desaturases (Fads2) with Δ6 and Δ5 activities have only a few aa differences and thus cluster closely together, as do rabbitfish Δ6/Δ5 and Δ4 desaturases. Thus, sequence and phylogenetic analyses are only indicators of whether desaturases are Fads1 or Fads2, but not of function. Although we have so far failed to isolate a Fads with Δ5 activity from *S. argus*, the feeding trial provided circumstantial evidence that LNA and LA can satisfy EFA requirements for normal growth and survival (unpublished data), which suggested *S. argus* may have the ability to synthesize LC-PUFA from C18 PUFA. Similarly, only Δ6 desaturase specificity was reported from the freshwater common carp Fads2, and this species also can effectively utilize VO (without LC-PUFA) to meet the essential fatty EFA requirement for normal growth and survival [11], [43]. However, as with *S. argus*, it is not clear whether or not other desaturases could be present in carp. Although both *S. argus* and rabbitfish are classed as marine herbivorous species, they are in fact euryhaline. Rabbitfish usually inhabit seawater but can tolerate salinity as low as 10 ppt [13], whereas *S. argus* can live in seawater, but normally inhabit estuaries, mangrove swamps, and even the lower reaches of rivers [18]. Therefore, the ecology of these “marine” teleosts, albeit similar, is different and may contribute to the apparent differences in LC-PUFA biosynthesis. Further research is required to elucidate the complete pathways and regulatory mechanisms of LC-PUFA biosynthesis in *S. argus*.

The expression of *S. argus fads2* was greatest in liver followed by brain, possibly reflecting the importance of fatty acid metabolism and LC-PUFA biosynthesis in these tissues [13], [44]. This is similar to the tissue expression pattern obtained in freshwater species like common carp [11], salmonids including rainbow trout [45], and Atlantic salmon [29], [46], [47], and the rabbitfish [14], in which the expression of a *fads2* was greatest in liver, followed by intestine/brain. In contrast, studies on marine fish showed that the expression of Δ6 desaturases was substantially higher in brain than all other tissues [33]–[36], [38], [48]. This has led to the hypothesis that the retention of Δ6 desaturase activity in marine teleost species has been to ensure sufficient 22:6n-3 in neural tissue through conversion of EPA via the so-called Sprecher shunt pathway [13].

Other than species differences, nutritional factors also affect LC-PUFA biosynthesis in teleosts through modulation of desaturase and elongase gene expression and subsequent effects on enzymatic activity. Many studies in freshwater and diadromous teleost fish species have shown that replacing FO by VO in diets resulted in increased expression of desaturase and, in some cases, elongase genes consistent with similarly increased enzymatic activity [29], [45]–[47], [49]–[53]. Similar results were also observed in marine fish [12], [33]. The results of the present study showed that dietary fatty acid composition affected the expression of *fads2* in *S. argus*, with higher expression levels detected in livers from fish fed diets with VO compared with the FO dietary group. As mentioned above, this was consistent with previous reports that replacing FO by VO resulted in increased Δ6 *fads* mRNA expression level in fish such as Atlantic salmon [29], [45], [46], [53], [54], Atlantic cod [33], gilthead seabream [55] and common carp [11]. Furthermore, a previous study also showed that the ratio of LNA/LA influenced the expression of key enzymes involved in LC-PUFA biosynthesis in rabbitfish [14]. The present results also showed that highest Δ6 *fads* mRNA expression was detected in liver of *S. argus* fed a diet with an LNA/LA ratio of around 1.72. As LNA and LA are substrates that compete for the Δ6 desaturase, this result may be related to the differential activity that the enzyme may have for these fatty acid substrates as suggested by the yeast expression assay.

However, the precise molecular mechanism involved in the nutritional modulation of LC-PUFA synthesis is unclear. Tocher et al [52] argued that a likely mechanism was based on classical feedback inhibition, with expression of LC-PUFA biosynthesis genes being suppressed by dietary LC-PUFA. In fish fed dietary VO, which lack LC-PUFA, this feedback inhibition is reduced and so suppression of gene expression is reduced leading to increased expression of the desaturase genes and consequently increased enzyme activity. Other authors have suggested that high levels of substrate C18 PUFA may also play a role as ligands involved in the activation of transcription factors that can enhance gene expression and thus promote the synthesis of LC-PUFA [56], [57]. Therefore, it appears that gene expression, fatty acid specificity, and dietary ratio of LNA/LA can combine to influence Δ6 Fads2 enzyme activity and that maximal efficiency of n-3 LC-PUFA biosynthesis could be obtained with particular levels of C18 PUFA [49], [55], [58]. This competition has been demonstrated as Eurasian perch showed higher ARA synthesis when the dietary ratio of LNA/LA was 0.64 than when it was 0.03 [59] and, consistent with this, an excess of 18:3n-3 inhibited Δ6 *fads* enzymatic activity and prevented the desaturation of 18:2n-6 in salmon [60]. Similarly, studies in salmonids showed that too high level of 18:2n-6 in VO may also inhibit the desaturation of 18:3n-3 [51], [61]. However, it has also been suggested that a dietary excess of 18:3n-3 could block Δ6 *fads* gene transcription [55]. Therefore, in addition to the LC-PUFA/C18 PUFA ratio, it is also important to maintain an appropriate LNA/LA ratio in diets to meet the requirements of essential fatty acids for normal life.

In conclusion, the present study has described the successful cloning and characterization of a *fads2* cDNA with Δ6 desaturase activity from the euryhaline herbivorous teleost *S. argus*. The predicted protein contained all the typical features of Fads-like proteins, and shared high homology with other teleost Fads. Highest mRNA expression level of *S. argus fads2* was detected in liver followed by brain, reflecting a pattern more associated with freshwater and salmonid fish than marine species. Replacing FO with a VO blend with a dietary ratio of LNA/LA of 1.72 resulted in greatest up-regulation of *fads2* expression in liver of *S. argus*. The results indicated that *S. argus* may have a different LC-PUFA biosynthetic pathway from rabbitfish although they share largely similar habitats and feeding habits.
